# Indiana

**Published:** 1897-02

**Authors:** 


*Indiana.*—At the present session of the State Legislature veterinarians will press for consideration a statute to regulate practice among the "Hoosier" veterinarians. Veterinarian Bitting is an active worker in the movement.

